# Updating Clinical Practices to Promote and Protect Human Milk and Breastfeeding in a COVID-19 Era

**DOI:** 10.3389/fped.2022.867540

**Published:** 2022-04-26

**Authors:** Johannes B. van Goudoever, Diane L. Spatz, Rebecca Hoban, Dani Dumitriu, Cynthia Gyamfi-Bannerman, Monika Berns, Liz McKechnie, Riccardo Davanzo

**Affiliations:** ^1^Emma Children's Hospital, Amsterdam University Medical Centers, Amsterdam, Netherlands; ^2^University of Pennsylvania School of Nursing & Children's Hospital of Philadelphia, Philadelphia, PA, United States; ^3^The Hospital for Sick Children (SickKids), University of Toronto, Toronto, ON, Canada; ^4^Columbia University Irving Medical Center, New York, NY, United States; ^5^Charité – Universitätsmedizin, Klink für Neonatologie, Berlin, Germany; ^6^Leeds Centre for Newborn Care, Leeds Teaching Hospitals, Leeds, United Kingdom; ^7^Institute for Maternal and Child Health Institute, IRCCS “Burlo Garofolo”, Trieste, Italy

**Keywords:** human milk, COVID-19, pandemic, breastfeeding, clinical practice

## Abstract

The COVID-19 pandemic has impacted breastfeeding and lactation globally, with clinical practices implemented early in the pandemic being mostly anti-breastfeeding, e.g., separation of mothers from their infants, and not evidence based. As the pandemic has progressed, evidence has emerged reconfirming the value of human milk and the importance of protecting and supporting breastfeeding, especially the initiation of lactation. However, it is clear that COVID-19 has changed the clinical care paradigm around breastfeeding and lactation support and, as such, it is imperative that practices adapt and evolve to maintain the emphasis on lactation support. We participated in a round table conference aiming to rescue and develop protocols and practices that support breastfeeding during the COVID-19 pandemic. One key area to target will be to maximize the use of the antenatal period. The early identification of lactation risk factors together with the development of person-centered methods to deliver breastfeeding information and education to parents-to-be will be critical. In addition, the establishment of a hospital culture that values breastfeeding and prioritizes the use of human milk will be integral for the motivation of health care professionals. That culture will also support active management of the initiation of lactation and the development of a 'back-up plan' toolkit to support the mother experiencing lactation difficulties. Post-discharge support will also be crucial with the development of both in-person and virtual lactation support programs, in particular for the immediate post-discharge period to benefit mothers who experience an early discharge process. These measures will allow for a new, adapted framework of practice that acknowledges the current COVID-19 paradigm and maintains the emphasis on the need to protect and support breastfeeding and the use of human milk.

## Introduction

The impact of COVID-19 on public health around the world has been profound. Efforts to mitigate the spread of SARS-CoV-2, the virus responsible for COVID-19 initially relied on non-pharmacological public health interventions (1, 2), although vaccine distribution is currently increasing dramatically (3). Unfortunately, the rapid introduction of these sweeping public health measures in response to the pandemic has severely jeopardized hospital and community based breastfeeding promotion programs and continues to do so in North America (4) as well as in Europe (5). The response by the research and medical communities has been swift, for example, there were over 6000 research articles focused solely on the impact of COVID-19 on maternal and child health and nutrition published in the 15 months from the start of the pandemic in February until April 30, 2021 (6). Of these articles, a notable proportion, ~10%, addressed the impact of COVID-19 on breastfeeding, human milk and infant feeding.

This response has reinforced the recognition of the use of human milk and breastfeeding as a major public health initiative in its own right. Indeed, the value of breastfeeding and human milk to public health has been well-documented (7). Despite this acknowledgment, it is important to note that prior to the COVID-19 pandemic, global breastfeeding rates still averaged only 41% (8). Accordingly, global health organizations such as the WHO, UNICEF and other non-governmental entities were already engaged in programs to improve breastfeeding initiation and duration rates worldwide. Therefore, the recent COVID-19-led recommendations that superseded and deprioritized practices known to promote and protect breastfeeding, such as skin-to-skin care, early initiation and rooming in, were particularly damaging to the support of lactation (9).

The introduction of extensive anti-breastfeeding practices early in the course of the pandemic was a consequence of the lack of information and preparedness in developing recommendations for pregnant and breastfeeding women and their infants (10). Of particular concern was the issue of vertical transmission between mother and infant. Interestingly, limited data from the 2003 SARS outbreak had shown no signs of vertical transmission (11–13) and no evidence of SARS in human milk (13, 14). Current data on SARS-CoV-2 and vertical transmission have agreed with the historical data, with no active virus being found in human milk (15) and very limited evidence of confirmed congenital SARS-CoV-2 infection (16, 17). Furthermore, both earlier (18) and more recent data (14, 19) have shown the presence of antibodies to both SARS-CoV-2, respectively, in human milk (20). In addition, antibodies have been found in human milk following vaccination of lactating women (21). As such, these data actually shift the perception of human milk away from being a risk to being a protective element for the newborn infant.

Despite these reassuring data, guidelines and practices that emerged early in the pandemic were largely contrary to the promotion and protection of breastfeeding (22). These practices, such as women going through labor and birth without the presence of a partner or support person (23); NICUs not allowing or restricting parental presence at the bedside (24); complete separation of mothers who were either confirmed or suspected as COVID-19 positive from their infants (25); discouragement or abandonment of skin-to-skin contact and direct breastfeeding (26); early discharge following birth (27) and a lack of access to in-person pediatric follow-up and breastfeeding assistance (4), are all recognized as providing a strong negative influence on the initiation of lactation and breastfeeding sustainability. Indeed, not only do these COVID-19-specific hospital practices, which were in opposition to the WHO recommendations (27), increase the risk of poor breastfeeding outcomes and are detrimental to the use of human milk, but they also can negatively impact maternal mental well-being (28).

As cautioned by Spatz et al. (22) the impact of these COVID-19-specific hospital practices, which were in opposition to the WHO recommendations (29), has been detrimental to breastfeeding and the use of human milk as well as maternal mental health (28). The negative influence of new hospital postnatal practices on the general population of new mothers has been clearly documented in a tertiary level maternity hospital in Italy, where exclusive breastfeeding rates at hospital discharge fell to 69.4% during lockdown compared to 97.7% during the pre-pandemic period (30). In this connection, ~18% of hospitals in a CDC-conducted survey of 1,344 hospitals in the USA reported a reduction in direct lactation support for mothers delivering in their hospitals. Notably, the impact of a reduction in lactation support in this manner affects both mothers with and without COVID-19 (31). Furthermore, ~73% of the surveyed hospitals discharged new mothers within 48 h. In all, ~12% of hospitals reported a decrease in breastfeeding rates (31).

Separation of the infant from the mother at birth was also shown to negatively correlate with breastfeeding at discharge (32, 33) and Bartick et al. (34) showed that 60% of mothers who were separated from their infants reported being 'very distressed', further highlighting the mental health ramifications to new mothers. This report was product of a Roundtable discussion to initiate a global dialogue on the impact of COVID-19 on breastfeeding and human milk practices.The aim of the current manuscript was to rescue and develop protocols and practices that support breastfeeding during the COVID-19 pandemic.

## Addressing the Clinical Need to Improve Breastfeeding Outcomes in the Covid-19 Era and Beyond

As the pandemic continues, there is the concern that this disruption to recognized quality standards of care will continue over the long term, as a sort of system inertia in hospitals (5), at the expense of practices known to promote and protect breastfeeding (22). Therefore, it is incumbent upon healthcare professionals to adapt to the current COVID-19 environment and conditions with respect to the development of protocols and practices that support breastfeeding. These protocols should be rescued and developed from the pre-pandemic era and appropriately integrated with the new science of coping with the risk of SARS-CoV-2 transmission (35).

One such opportunity is to maximize the use of the antenatal care period to provide parents tailored breastfeeding information. The current antenatal paradigm is neither allowing all families to make an informed feeding decision nor is it effectively preparing families about the physiology of lactation and the science of human milk (36). Indeed, it has been suggested that antenatal care is somewhat overdue to be redesigned (37). The development of standardized programs relating to antenatal breastfeeding education have been shown to improve breastfeeding intention (38, 39). Furthermore, in a systematic review of interventions for promoting the initiation of breastfeeding, informal one-to-one, needs-based education delivered by a healthcare professional in the antenatal period was found to be an effective means of increasing breastfeeding initiation (40). This was further confirmed in relation to mothers' direct perceptions of an antenatal program, where new mothers suggested that an individualized approach could improve the delivery of information (41).

Whilst it is clear that antenatal support is critical, the way information is provided is also important. One-on-one and face-to-face support is favored due to its ability to deliver both practical advice as well as emotional support. Indeed, person-centered, emotional care may be as important as the educational content of a breastfeeding support program (42). However, the COVID-19 pandemic with its multiple lockdown episodes resulted in a reduction of face-to-face professional and peer support (43) although this has been at least partially addressed by the rapid implementation of telemedicine services (44). Indeed, there is growing evidence for the embracing of telemedicine methodologies (45), including for breastfeeding support (43, 46, 47). Interestingly, online education and smartphone applications, together known as Mobile health, are largely acceptable to most marginalized populations (48) and therefore may actually provide a means of bridging the equity gap if technological resources are provided.

A positive outcome from the optimization of antenatal care is the development of a comprehensive breastfeeding plan. Such a plan forms the intersection between maternal education/readiness and facility preparedness (i.e., the implementation of breastfeeding promoting protocols and processes), with respect to the early postpartum support of breastfeeding. In this regard, it is important that hospital practices and protocols take into account maternal lactation risk factors known to impact the initiation of lactation, e.g., preterm birth, gestational diabetes, maternal obesity, cesarean section and primiparity, mother-infant separation, and that these are included in the breastfeeding plan (49–57). These factors, when managed with practices known to be positive for the initiation of lactation, i.e., informed decision making, initiation and maintenance of milk supply, skin-to-skin care, and direct breastfeeding (22), will allow for an increase in the rates of human milk usage at discharge (58–60).

Perhaps now, more than previously, it is important for healthcare professionals to acknowledge the value of such antenatal planning as well as staff education related to policy and protocol development. Insufficient maternal milk volume can have its origins in the first week postpartum, the decisive period during which the mammary gland undergoes programming processes that regulate long-term milk synthesis (61–63). As such, it is important to establish an institutional culture that prioritizes the use of human milk (64) to facilitate the implementation of interventions supporting breastfeeding. Engagement of health care professionals via quality improvement (QI) programs has been shown to be one way to address institutional barriers to human milk usage; indeed staff education and motivation is one QI initiative that can be used to obtain stakeholder buy in (65), as is a multidisciplinary approach (66).

Along with the improvements to antenatal education and facility preparedness, it is important that both the mother and the healthcare professional acknowledge that the initiation of lactation requires active management in the postnatal period. Evidence-based protocols and programs dedicated to the initiation and maintenance of lactation are well-established and have been shown to improve breastfeeding outcomes (58, 67). As such, it will be the adaptation of those known protocols to fit within post COVID-19 institutional restrictions that will be most critical. For example, the steps recommended to promote the initiation of lactation, such as early and frequent breastfeeding, non-nutritive sucking, skin-to-skin care, and assessment of milk transfer are all impacted in the case of decreased direct postpartum lactation support and accelerated discharge (31) as well as if the mother and infant are separated (25). In this regard, it is important to emphasize that known or suspected infection of SARS-CoV-2 of a mother does not necessitate the automatic separation of mother and infant (27, 35). This should also not preclude the use of any of these evidence-based practices known to improve the initiation of lactation (27, 68, 69), especially since robust and specific antibodies against SARS-CoV-2 have been detected in human milk produced by women with COVID-19 (14, 19).

In addition to the development of adaptive protocols as a first line to protect and promote breastfeeding and lactation, it is also important to adjust any anticipatory guidance to the same COVID-19 circumstances. For mothers with known risk factors to initiation, such as preterm birth, gestational diabetes mellitus, cesarean section and primiparity (49–52, 55–57), or those who experience difficulties during the initiation stage, the second line-of-action interventions also need to be viewed using a COVID-19 lens. In these instances, the use of a breast pump to support mothers with lactation initiation has been well-documented (67, 70). It is therefore important to also address breast pump usage requirements within a COVID-19 framework (69), particularly the establishment of dedicated protocols to facilitate pump set up as well as cleaning. Furthermore, the requirements of the mother during the milk expression process, e.g., hand hygiene, use of a mask, also need to be explicit. Lastly, recognized protocols for the storage and use of the collected milk also need to be set in place.

In conjunction with the adaptation of in-hospital care practices to protect and support lactation, attention must also be given to post-discharge care. As discharge timeframes have shortened significantly (31), although not universally (5) and lactation support staff and education has been reduced (22), post discharge clinical support for breastfeeding is more important than ever before. Of particular importance is the immediate post discharge period, especially considering the Academy of Breastfeeding current recommendation that all breastfed infants be seen by a healthcare provider at 3–5 days of life or within 48–72 h of discharge (71). However, as a consequence of COVID-19 restrictions, many mothers lack face-to-face support in the post-discharge phase (43).

At hospital discharge, health professionals should provide new families with comprehensive information on the resources available in the community, congruent with Step 10 of the Baby Friendly Hospital Initiative (72). Similar to antenatal support, telehealth or virtual strategies are one way of bridging the post-discharge follow-up gap and may be a way of creating an “on-call, 24/7” support service for new mothers. In this regard, it has been recommended that in place of in-person visits, breastfeeding support should be provided via telehealth services (73). Indeed, the use of virtual strategies to improve breastfeeding outcomes has been shown to be positive (74) can work in both large class as well as small group formats (75) and can also be incorporated in a pediatric outpatient setting (76). Additionally, specific post-discharge challenges like latch difficulties may be better addressed in-person. However, it should be recognized that despite these encouraging outcomes, some mothers still have mixed feelings in relation to telehealth services and desire the emotional care that comes from in-person support from family, friends and lactation healthcare professionals (43, 77).

## Conclusion

**Figure 1:**
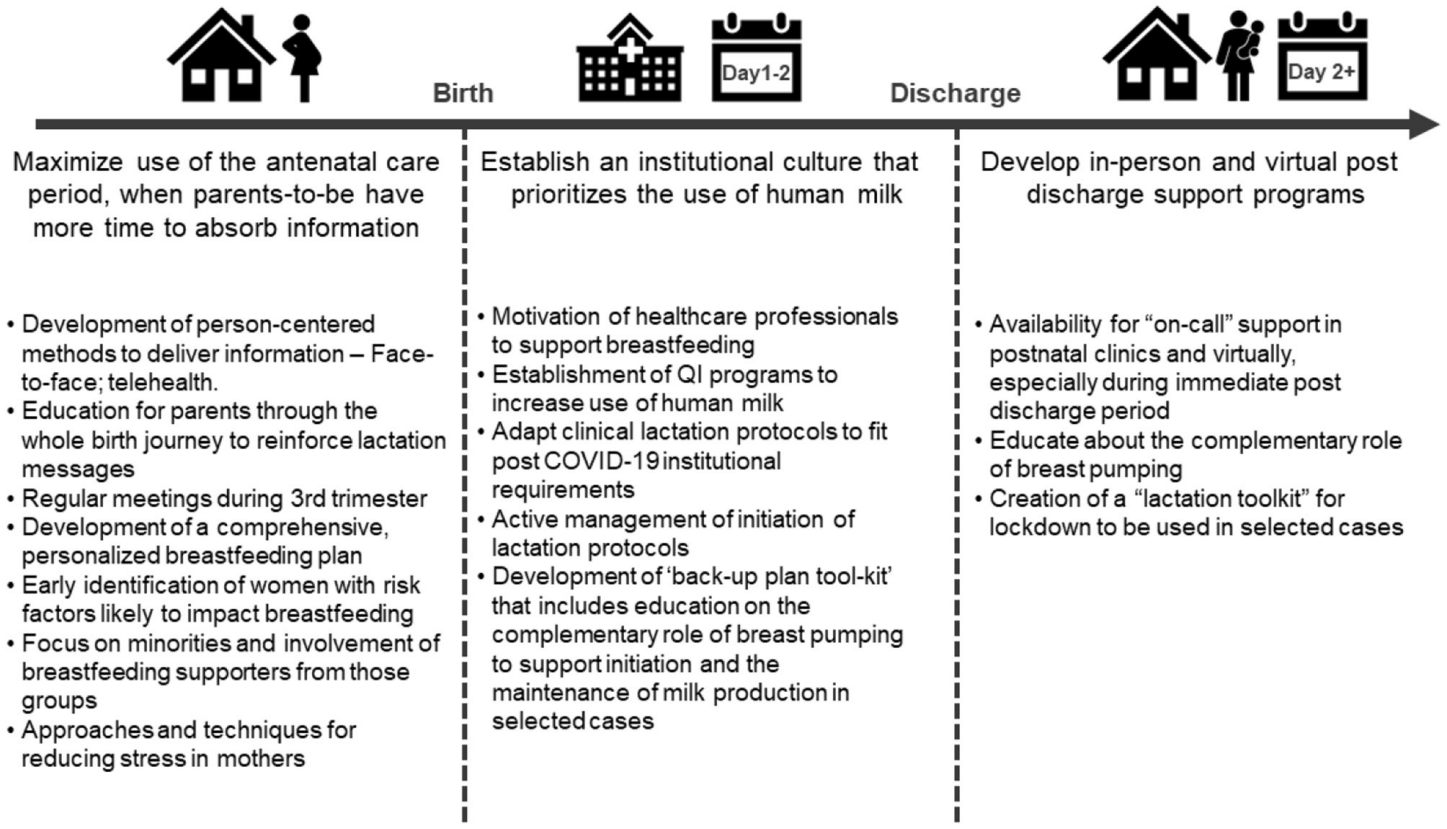
Target clinical practice areas to address in order to maintain the promotion and protection of human milk and breastfeeding in a post COVID-19 era.

The current pandemic has provided a tremendous impact on global public health measures. In particular, the impact on breastfeeding has been significant with several reports documenting how breastfeeding promotion and support services have been jeopardized by specific changes to both hospital and public health practices (22, 25, 26). Given the acknowledgment of this new COVID-19 paradigm, it is crucial that healthcare providers pivot to develop a new breastfeeding support 'roadmap to action' that acknowledges recognized standards of care and updates clinical practice whilst taking into account the current, and most likely long-term, requirements that are a consequence of the changes brought about by COVID-19 (Figure 1). It is strongly suggested that healthcare professionals take ownership of the antenatal care phase and maximize this period when parents-to-be have more time to absorb and process information. By engaging with parents-to-be in this phase, there is an opportunity to educate them on the importance of the initiation of lactation and to develop a holistic breastfeeding plan that works within the hospital breastfeeding initiation protocols. In addition to the development of a new mother's breastfeeding plan, it is important that the role of the healthcare professional is adapted to ensure that not only a motivation to actively support breastfeeding initiation exists, but that suitable 'back-up' plans and protocols are developed as well, including the use of a breast pump to support initiation for those mothers with selected lactation difficulties/risk factors or who meet with lactation challenges. Lastly, in response to accelerated discharge protocols, there is a need to remodel post discharge support, in particular to embrace new modalities of interaction with mothers, whilst taking into account both their breastfeeding and mental health requirements. These measures provide a new, adapted framework of practice that acknowledges the current COVID-19 paradigm and maintains the emphasis on the need to protect and support breastfeeding and the use of human milk.

## Author Contributions

All authors listed have made a substantial, direct, and intellectual contribution to the work and approved it for publication.

## Conflict of Interest

JG serves as member of the National Health Council and is also director of the Dutch National Human Milk Bank. The remaining authors declare that the research was conducted in the absence of any commercial or financial relationships that could be construed as a potential conflict of interest.

## Publisher's Note

All claims expressed in this article are solely those of the authors and do not necessarily represent those of their affiliated organizations, or those of the publisher, the editors and the reviewers. Any product that may be evaluated in this article, or claim that may be made by its manufacturer, is not guaranteed or endorsed by the publisher.
